# Combination Immunotherapy Approaches for Pancreatic Cancer Treatment

**DOI:** 10.1155/2018/6240467

**Published:** 2018-03-07

**Authors:** Xianliang Cheng, Gang Zhao, Yunqi Zhao

**Affiliations:** ^1^School of Pharmaceutical Sciences & Yunnan Provincial Key Laboratory of Pharmacology for Natural Products, Kunming Medical University, Kunming, Yunnan, China; ^2^The First Affiliated Hospital of Dalian Medical University, Dalian, Liaoning, China

## Abstract

Pancreatic ductal adenocarcinoma is a lethal malignant disease with a very low medium survival. Currently, metastatic pancreatic cancer poorly responds to conventional treatments and exhibits an acute resistance to most chemotherapy. Few approaches have been shown to be effective for metastatic pancreatic cancer treatment. Novel therapeutic approaches to treat patients with pancreatic adenocarcinoma are in great demand. Last decades, immunotherapies have been evaluated in clinical trials and received great success in many types of cancers. However, it has very limited success in treating pancreatic cancer. As pancreatic cancer poorly responds to many single immunotherapeutic agents, combination immunotherapy was introduced to improve efficacy. The combination therapies hold great promise for enhancing immune responses to achieve better therapeutic effects. This review summarizes the existing and potential combination immunotherapies for the treatment of pancreatic cancer.

## 1. Introduction

Pancreatic ductal adenocarcinoma (PDAC) is the most common form of pancreatic cancer (approximately 90%), and it is the third leading cause of cancer death with an overall 5-year survival rate of 5–10% [1, 2]. Since PDAC is normally diagnosed at a late stage, the majority of patients with PDAC do not survive a year after diagnosis. The standard chemotherapy for metastatic PDAC is FOLFIRINOX, a combination of oxaliplatin, irinotecan, fluorouracil, and leucovorin [3]. However, concerns for toxicity and adverse side effects quickly restricted patients to the treatment. Due to rising incidence of PDAC, there is a major unmet need to develop novel promising therapeutic strategies.

Immunotherapy opens a new era in cancer treatment. People achieve great success in cancer vaccines and immunomodulators, such as checkpoint blockade to induce endogenous host immune response. Nevertheless, PDAC has nonimmunogenic and immune-suppressive microenvironment, and immune checkpoint inhibitor monotherapy alone lacks efficacy in this disease. Tremendous efforts have been made to seek a new strategy to improve immunotherapy efficacy.

Innate immune cells express pattern-recognition receptors, such as dectin-1, on their surfaces. Dectin-1 was found highly expressed in human PDAC tumor and peritumoral inflammatory compartments. The dectin-1 signal transduction pathway opens a new area in the anticancer therapeutic application. It could be an attractive target for PDAC immunotherapy.

Adoptive immunotherapy utilizing chimeric antigen receptor-engineered T-cells is being exploited as a promising strategy to redirect patient's T-cells against tumors and reduce tumor load. Several antigens, such as carcinoembryonic antigen and mesothelin, have been chosen as the target of the engineered T-cells. Researches indicate that this strategy showed encouraging results. However, serious adverse events were associated with the treatment [4, 5] such as cytokine release syndrome and neurological toxicity [6]. Other therapeutic approaches need to be done to solve the safety issue.

In this review, we summarized recent findings in the development of novel combination immunotherapies to improve treatment efficacy in PDAC.

## 2. Immune Checkpoint

Immune checkpoints are involved in regulation of antigen recognition of T-cell receptor by costimulatory or inhibitory signaling transduction in the immune system. Immune checkpoint blockade therapy achieves great success in treating many types of cancers [7]. It targets T-cell regulatory pathways to enhance anticancer immune response. Since the immune response has dynamic nature, research indicates that combination therapies may provide a better survival benefit for cancer patients [8].

### 2.1. Cytotoxic T-Lymphocyte-Associated Antigen-4 (CTLA-4)

T-cell exclusion is obviously evident in PDAC, in which effector T-cells are often scarce within tumor tissue and confined to peritumoral lymph nodes and lymphoid aggregates [9]. CTLA-4 is an immune checkpoint receptor expressed on regulatory T (Treg) cells and recently activated conventional T-cells [10]. It is a negative regulator of T-cell activation, and it is also known as CD152. CTLA-4 is homologous to CD28 and they share the same ligands. Both B7-1 (CD80) and B7-2 (CD86) ligands are expressed on antigen-presenting cells (APCs) and can render costimulatory signals to T-cells. Upon activation, T-cells express CTLA-4 on the cell surface. CTLA-4 engagement with B7 inhibits T-cell activation. CTLA-4 has higher affinity to B7 ligands compared to CD28. CTLA-4 ligation delivers an inhibitory signal to T-cells, whereas CD28 delivers a stimulatory signal [11, 12]. The anti-CTLA-4 antibody can blockade CTLA-4 interaction with B7 and prevents the inhibitory signal [13]. Targeting CTLA-4 with a human anti-CTLA-4 antibody has demonstrated therapeutic success in the treatment of melanoma [14]. Then blockade of CTLA-4 may be a promising new approach to cancer therapy and constitutes a novel approach to induce host responses against tumors. It could downregulate the immune system and produce durable anticancer responses [15]. However, there is no sufficient evidence showing that CTLA-4 is a potential therapeutic target for PDAC immunotherapy [16]. Little benefit has been achieved so far by applying CTLA-4 antibodies alone in PDAC treatment. This might be due to high tumor burden and the intrinsic nonimmunogenic nature of pancreatic cancer that cause immune quiescent, and the blockage of only one checkpoint is not enough for immunosuppressive reduction.

Ipilimumab (MDX-010) is a fully humanized IgG1 monoclonal antibody that works by blocking the ligand-receptor interaction of B7-1/B7-2 and CTLA-4. Thereby, ipilimumab has the potential to increase antigen-specific immune responses. In 2011, it is approved by US Food and Drug Administration (FDA) to treat metastatic melanoma [17], and the thread name is Yervoy. Ipilimumab, as a single agent, has been tested in PDAC patients. Despite the fact that ipilimumab at a dose of 3.0 mg/kg was minimally effective for the treatment of advanced pancreatic cancer, the delayed response case suggests that it deserves further investigation and complete assessment of immunotherapeutic approaches to pancreatic cancer [18]. Therefore, the concept of synergy between immune checkpoint blockade and cancer vaccines was brought up. It has shown encouraging results in PDAC in treatment combinations with granulocyte macrophage-colony stimulating factor (GM-CSF) cell-based vaccines (GVAX). In a phase I b trial study, ipilimumab 10 mg/kg + GVAX treatment group showed prolonged median overall survival and 1-year overall survival compared to ipilimumab 10 mg/kg treatment group. It indicates that checkpoint blockade in combination with GVAX has clinical benefit potential for PDAC patients [19].

Tremelimumab (CP 675206; CP-675; CP-675,206; CP-675206; Ticilimumab) is fully humanized IgG2 monoclonal antibody that antagonizes CTLA-4. It has been used for the treatment of various cancers, such as melanoma, colorectal cancer, prostate cancer, and pancreatic cancer [20]. A phase I study of tremelimumab combined with gemcitabine to treat pancreatic cancer was performed [21]. The study demonstrated a safe and tolerable profile and suggested that anti-CTLA-4 antibody in combination with standard chemotherapy might provide synergistic anticancer activity without increasing side effects.

### 2.2. Programmed Death 1 (PD-1)

PD-1 protein, known as another immune checkpoint, is expressed on the surface of activated T-cells and is associated with programmed cell death [22]. PD-1, together with one of its ligands, programmed death-ligand 1 (PD-L1; also called B7-H1 or CD274), a B-7 family ligand, can suppress the overstimulation of immune responses and commit to the maintenance of immune tolerance to self-antigens [23]. It is an immunosuppressive pathway that is upregulated in tumor cells. PD-L1 is expressed by immune cells and various cancer cells, including breast, cervical, colorectal, gastric, glioblastoma, melanoma, non-small-cell lung, ovarian, pancreatic, and urothelial cancer [24]. Binding of PD-1 to its ligands inhibits T-cell activity and restricts tumor cell killing [25–28], leading to detrimental immune responses and preventing autoimmunity [29]. Blocking the ligation between PD-1 and PD-L1 should, therefore, augment immune response* in vitro* and initiate antitumor activity in preclinical models [30–32]. Because of this, targeting PD-1/PD-L1, as immune checkpoint blockade, has been developing in oncologic therapy for various cancers as of late. For instance, pembrolizumab (trade name Keytruda, 2014), nivolumab (trade name Opdivo, 2014), atezolizumab (trade name Tecentriq, 2016), and Durvalumab (trade name Imfinzi, 2017) were approved by US FDA for the treatment of metastatic non-small cell lung cancer, squamous cell carcinoma of the head and neck, metastatic melanoma, and bladder cancer, respectively. However, in the treatment of pancreatic cancer, there tends to be no apparent therapeutic effects of a single antibody [33, 34].

Therefore, to overcome the resistance of anti-PD-1/PD-L1 monotherapy, combination therapy strategies have been suggested for PDAC treatment. The current study shows that 92% clinical efficacy rate can be achieved when pembrolizumab is combined with gemcitabine plus nab-paclitaxel [35]. Meanwhile, combining radiotherapy with PD-1/PD-L1 blockade therapy could increase radiosensitization and enhance the tumor cell immunogenicity [36]. Anti-PD-1/PD-L1 also can be combined with targeted therapies. Research indicates that the combination with poly (ADP-ribose) polymerase (PARP) inhibitors may be effective against pancreatic cancer with BRCA1/2 mutations [37]. Since many mechanisms are involved in immunosuppression of PDAC, two different immunotherapies also can be combined. When anti-PD-1 combined with GVAX, the murine survival rate was significantly improved compared to anti-PD-1 or GVAX monotherapy [38].

Therefore, as discussed above, the combination therapy of anti-PD-1/PD-L1 may overcome the immune resistance properties of PDAC and could improve the therapeutic efficacy of anti-PD-1/PD-L1 immunotherapy.

## 3. Dectin-1 and Innate Immune System

Dectin-1, also known as C-type lectin domain family 7 member A, is encoded by* CLEC7A *gene and is a pattern-recognition receptor expressed by myeloid-monocytic lineage cells [39]. Dectin-1 recognizes *β*-glucans polysaccharides in fungal cell walls and is directly associated with the innate immune system [40]. Dectin-1 was found highly expressed in both mouse and human PDAC tumors and macrophages. Ligation of dectin-1 with galectin-9, a member of the *β*-galactoside-binding family of lectins and a functional ligand for dectin-1, can accelerate the progression of PDAC in mice. The treatment by dectin-1 agonist could induce accelerated PDAC progression.

Galectin-9 is also overexpressed in both murine and human PDAC and upregulated in diverse PDAC-infiltrating myeloid cells and cancer cells. The blockade of galectin-9 could extend animal survival. Similarly, elevated galectin-9 expression associated with reduced survival in human PDAC [41]. The ligation of dectin-1 with galectin-9 in pancreatic cancer can cause mouse and human tolerogenic macrophages programming and adaptive immune suppression. The upregulated expression of either dectin-1 or galectin-9 plays a pivotal role in the ability of pancreatic tumor cells to evade the host's immune system, causing immunotherapy failure. Due to animal survival experiments and the limitation of targeting dectin-1 or galectin-9, additional treatments are required for the immunotherapy [41]. Therefore, immunotherapy regimen targeting PD-1 has been suggested to combine with therapies targeting either dectin-1 or galectin-9. This strategy might offer synergistic efficacy for cancer treatment.

## 4. Chimeric Antigen Receptors (CARs) and Adoptive Immune System

The better understanding of T-cell biology and genetic engineering allows us to modify T-cells by associating a synthetic molecule and infusing them into tumor tissue to enhance the immune response against malignant lesion [42]. Genetically engineered T-cells can specifically target cancer cells to eradicate tumor burden through a T-cell receptor or chimeric antigen receptors (CARs). CARs, also known as chimeric immunoreceptors, are engineered recombinant receptors with an intracellular signaling domain consisting of T-cell receptor-CD3-*ζ* domain and an extracellular single-chain variable antibody fragment [43]. CARs can directly bind to tumor-associated antigens, carbohydrates or glycolipids.

In August 2017, US FDA has approved a CARs therapy (tisagenlecleucel, Kymriah, Novartis) that used adoptive cell transfer technique to treat acute lymphoblastic leukemia. Actually, the CARs therapy technology was first introduced in 1989. The first generation of CARs, the targeting moiety, is coupled to a CD3-*ζ* module, which initiates T-cell activation and enables T-cell to mediate cytotoxicity [44]. This generation was shown clinically ineffective in patients with diverse solid tumors [45]. The second generation of CARs incorporate an additional costimulatory domains (CD28 or CD137), which have enhanced T-cell proliferation as well as cytotoxic activity [46]. The costimulatory effect may be imparted by receptors, for example, 4-1BB [47], CD28 [48], or ICOS [49]. A complete response of 90% was achieved in lymphodepleted patients treated with the second-generation CAR T-cells [50]. The third generation of CAR comprises CD3-*ζ* and two additional costimulatory signaling domains, CD28 and 4-1BB, or CD28 and OX40 [51]. T-cell targeting by a TCR faces a challenge because it is restricted by human leukocyte antigen (HLA) while CARs help T-cells to target tumor cells directly and are not restricted by HLA [52]. Both second- and third-generation CARs have shown preclinical efficacy in mesothelioma and ovarian xenograft models [53]. However, there is still a lot to learn about which method will be the safest and best suited to treat solid tumors [54].

The antigens overexpressed on solid tumor cells but with limited or no expression on normal cells can be promising targets for CAR T-cell therapy. Pancreatic cancer exhibits a number of tumor-specific antigens, such as carcinoembryonic antigen, mesothelin, HER-2, and MUC1, which are promising applicants for testing CARs T-cell therapy [55, 56].

### 4.1. Carcinoembryonic Antigen (CEA)

CEA is a set of glycoproteins involved in cell adhesion, and the expression of CEA is low in healthy adults. The serum level of CEA can be elevated in some types of a cancer patient; for example, the antigen expressed in pancreatic adenocarcinomas is nearly 75% [57, 58]. CEA can be recognized by CARs T-cells, which makes it a valuable candidate target in CARs T-cell therapy for pancreatic cancer. In addition, the CEA level in serum can be a specific marker used routinely to monitor disease progression and tumor load. It was found that when patients received the highest dose of anti-CEA CARs T-cell, the levels of CEA declined [59]. In a clinically relevant model in murine, adoptive transfer of anti-CEA CAR-engineered T-cells was able to specifically and efficiently reduce the size of pancreatic tumors below the limit of detection in all mice and give continuing tumor eradication in 67% of mice [60]. This suggests the notion that CAR T-cells targeting CEA have the potential to treat pancreatic cancer.

### 4.2. Mesothelin (MSLN)

MSLN is a glycosyl-phosphatidylinositol- (GPI-) linked membrane glycoprotein, which is highly expressed in mesothelioma, pancreatic, lung, ovarian, and other cancers, but lowly expressed in normal tissue [61]. The aberrant expression of MSLN involves the aggressiveness and transformation of tumors through promoting cancer cell proliferation [62]. In a preclinical model, T-cells were engineered to express an affinity-enhanced TCR and were utilized to target MSLN antigen in a genetically engineered model of autochthonous PDAC. Engineered T-cells are known to accumulate in PDAC, inducing tumor death and stromal remodeling. Engineered human T-cells lyse PDAC cells in vitro, which further supports TCR-based strategy for the treatment of PDAC [63]. Other similar studies of CAR T-cells targeting MUC1 [64], CD24 [65], and HER2 [65] were tested, leading to tumor regression in mice. MORAb-009 is a chimeric antimesothelin monoclonal antibody that was utilized to target tumor-associated mesothelin overexpressed on pancreatic, ovarian, lung, and colorectal carcinoma. It was found that MORAb-009 reduced tumor growth in mesothelin-positive cancers and enhanced effectiveness of chemotherapy [66]. MORAb-009 was found to be safe in phase I clinical study of 24 mesothelin-positive patients, which included 7 pancreatic cancer patients [67].

### 4.3. Treatment Concerns

The treatment of pancreatic cancer through CARs T-cell therapy still remains challenging because of on-target and off-tumor effects. Identification of choosing an ideal tumor-restricted antigen is rare and rigorous. The CAR T-cell has toxicities to healthy tissue, causing trials cease, especially when the target tissue is expressed in central tissues, such as lungs, heart, or liver [6]. This has once again illustrated the importance of careful target antigen selection.

Cytokine release syndrome (CRS) and neurological toxicity are the most common severe side effects of CARs T-cell immunotherapy, and tocilizumab (IL-6 blocker) is used to treat patients with CRS. CRS correlates with disease burden [68] and is potentially due to the release of inflammatory cytokines produced by amounts of activated CARs T-cells [69].

Serious adverse events of CARs T-cells immunotherapy can be related to several factors. The current approaches of toxicity management have incorporated dual targeting strategies [70]. Suicide gene combined with cellular therapeutic products can eliminate the majority of CARs modified T-cells and prevent contiguous cells and/or tissues from collateral damage. This strategy has been advised to be combined together to reduce the side effects.

Another approach is to aim at coexpressing “inhibitory” CARs (iCARs) to avoid normal tissue targeting. iCARs can incorporate with CTLA-4 and PD-1 domain to transmit an inhibition signal instead of an activation signal [71]. Targeting multiple cancer-specific markers simultaneously could result in increased specificity and better therapeutic efficacy. “TanCAR” was introduced to avoid the off-target effect. TanCar mediates bispecific activation and targets to T-cells. This strategy could potentially offer a safer approach to minimize the severe adverse effects [72].

## 5. Summary

Pancreatic cancer remains a devastating lethal disease with poor prognosis. Immunotherapy uses the self-immune system to fight cancer and is emerging as the fourth pillar of cancer treatment, after surgery, chemotherapy, and radiation therapy. In the immune system, immune checkpoints are molecules involved in cell signaling transduction. By inhibiting T-cell signaling, many cancers can evade from the immune system elimination. Immune checkpoint therapy can target T-cells' regulatory pathways and enhance antitumor immune responses. Immune checkpoint inhibitors, such as anti-CTLA-4, anti-PD-1, and anti-PD-L1, can enhance antitumor immunity and mediate cancer regressions in many types of cancers. These findings have established immune checkpoint blockade immunotherapy as a viable treatment option for patients with advanced cancers. However, due to pancreatic cancer's unique characteristics, the treatment of single immune checkpoint inhibitor, anti-CTLA-4, anti-PD-1, or anti-PD-L1, has shown minimal clinical benefits in the treatment of advanced PDAC. Although some early success has been achieved with monotherapies blocking PD-1 pathways, the efficacy of an anti-PD-1/PD-L1 monotherapy may be ineffective when treating pancreatic cancer in an immune system suppressed by high tumor burden and intrinsic nonimmunogenic nature [73]. However, preclinical models have indicated that combinatorial approaches will provide some favorable clinical outcomes. Therefore, combination immune therapy that targets PDAC immune checkpoints is currently the subject of intense study.

Besides, antibody blockers of novel immune checkpoints, which may be effective if employed in treatment combinations, are under development. These include lymphocyte activation gene 3 (LAG-3), killer inhibitory receptors (KIRs), B7-H3 (CD276), T-cell immunoglobulin and mucin-3 (TIM-3), V-domain Ig-containing suppressor of T-cell activation (VISTA), T-cell immunoglobulin and immunotyrosine inhibitory motif (ITIM) domain (TIGIT), and indoleamine 2,3-dioxygenase (IDO) [7]. The blockade of these checkpoints can be combined with anti-PD-1 or anti-PD-L1 to enhance antitumor immunity. Both innate and adaptive immunity are cooperating to promote tumor progression in PDAC. Dectin-1 plays a role in the innate immune response. The ligation of dectin-1 with galectin-9 in PDAC results in tolerogenic macrophage programming and adaptive immune suppression. The development of the therapeutics that target dectin-1/galectin-9 axis in combination with other immunotherapies will potentially be an attractive strategy for immunotherapeutic for human PDAC.

Adoptive immunotherapy using CARs T-cells is emerging as a novel approach to pancreatic cancer immunotherapy. Despite the improvement of CAR T-cells therapy within the last decade, its application as a treatment still remains in its infancy for PDAC. There are many obstacles in the clinical development of CARs-based immunotherapy for PDAC, such as significant toxicity profile and high cost. To ensure safety, a combination of two targets has been applied and will be investigated in clinical trials. Therefore, the selection of suitable targets to increase the precision of tumor targeting is crucial in future CARs development.

Immunotherapy has been successfully applied in treating various types of cancers. It also has the potential to treat pancreatic cancer. Even though PDAC does not have a good response to many single immune therapeutic agents, such as immune checkpoint inhibitors, combination therapy opens new possibilities. Therefore, more preclinical and clinical studies are needed to further identify better combination immunotherapy for PDAC.
